# Opening the archives for state of the art tumour genetic research: sample processing for array-CGH using decalcified, formalin-fixed, paraffin-embedded tissue-derived DNA samples

**DOI:** 10.1186/1756-0500-4-1

**Published:** 2011-01-04

**Authors:** Danielle de Jong, Sofie LJ Verbeke, Danielle Meijer, Pancras CW Hogendoorn, Judith VMG Bovee, Károly Szuhai

**Affiliations:** 1 Department of Molecular Cell Biology, Leiden University Medical Center, Einthovenweg 20, 2333ZC Leiden, The Netherlands; 2 Department of Pathology, Leiden University Medical Center, Albinusdreef 2, 2333ZA Leiden, The Netherlands

## Abstract

**Background:**

Molecular genetic studies on rare tumour entities, such as bone tumours, often require the use of decalcified, formalin-fixed, paraffin-embedded tissue (dFFPE) samples. Regardless of which decalcification procedure is used, this introduces a vast breakdown of DNA that precludes the possibility of further molecular genetic testing. We set out to establish a robust protocol that would overcome these intrinsic hurdles for bone tumour research.

**Findings:**

The goal of our study was to establish a protocol, using a modified DNA isolation procedure and quality controls, to select decalcified samples suitable for array-CGH testing. Archival paraffin blocks were obtained from 9 different pathology departments throughout Europe, using different fixation, embedding and decalcification procedures, in order to preclude a bias for certain lab protocols. Isolated DNA samples were subjected to direct chemical labelling and enzymatic labelling systems and were hybridised on a high resolution oligonucleotide chip containing 44,000 reporter elements.

Genomic alterations (gains and losses) were readily detected in most of the samples analysed. For example, both homozygous deletions of 0.6 Mb and high level of amplifications of 0.7 Mb were identified.

**Conclusions:**

We established a robust protocol for molecular genetic testing of dFFPE derived DNA, irrespective of fixation, decalcification or sample type used. This approach may greatly facilitate further genetic testing on rare tumour entities where archival decalcified, formalin fixed samples are the only source.

## Background

The introduction of high-throughput, high-resolution molecular screening tools had tremendous impact on molecular genetic studies both for constitutional and tumour genetic investigations [1,2]. Whilst the accessibility of good quality samples for constitutional genetic studies is often achievable, for cancer genetic investigations it has remained a hurdle especially for those dealing with rare tumour entities. A comprehensive study of rare cancers, such as bone tumours, requires the use of archived tissue materials such as formalin fixed paraffin embedded tissue (FFPE) [3-5]. It is well known that the quality of FPPE-derived DNA is both fixation time- and fixative-dependent and is highly variable between different institutions. 10% buffered formalin is a commonly used fixative in routine diagnostic labs. Long term storage of this fixative leads to the formation of formic acid and methanol by the Cannizzaro-reaction. Formic acid promotes the breakdown of the DNA and thus inferior quality of DNA is extracted from these tissue samples. To process bone derived tumour samples, an extra decalcification step is necessary to remove the Ca^2+^-containing matrix part of the tissue. This can be achieved either by EDTA treatment or by an extensive formic acid treatment. EDTA treatment is a labour-intensive procedure and takes up to several weeks of incubation. The treatment introduces limited breakdown of DNA but because of its lengthy procedure it is impractical for routine diagnostics. The formic acid-based decalcification procedure introduces a tremendous breakdown of DNA within these samples. As a result, most of these samples are usually regarded as unsuited for molecular biological testing. The formic acid-based decalcification has been the gold standard procedure at many institutions, meaning that most of the archival material collected from multiple sources has been treated in this way. The goal of our study was to establish a modified DNA isolation protocol with quality controls enabling array-CGH testing on decalcified samples irrespective of fixation and decalcification steps used. Isolated DNA samples were labelled using two FFPE labelling kit systems and were hybridised on a high resolution oligonucleotide chip containing 44k reporter elements.

## Materials and methods

### Sample selection

Samples were selected for molecular cytogenetic testing from various partner institutions within the EuroBoNet consortium http://www.eurobonet.eu for different projects (rare chondrosarcoma subtypes of bone and primary angiosarcoma of bone) dealing with decalcified FFPE (dFFPE) samples. Samples used in this study represent both tumours with high cellularity and a low extracellular matrix proportion as well as samples with low cellularity and an excessive extracellular matrix composition. Sample collection dates varied from 1990 until 2008. Samples were all fixed in 10% buffered formalin but the exact fixation times and conditions are not known (Table 1).

**Table 1:** Overview of samples included in this study

Sample ID	Diagnosis	Collection date	Material	Cellularity	Extracellular matrix	Decalcification
1	Rare chondrosarcoma	2004	dFFPE	High	Low	Formic acid

2	Rare chondrosarcoma	2007	dFFPE	High	Low	Formic acid

3	Rare chondrosarcoma	2007	dFFPE	Low	High	Formic acid

4	Rare chondrosarcoma	1996	dFFPE	Low	High	Formic acid

5	Rare chondrosarcoma	1997	dFFPE	High	Low	Formic acid

6	Rare chondrosarcoma	2005	dFFPE	Low	High	Formic acid

7	Rare chondrosarcoma	2005	dFFPE	High	Low	Formic acid

8	Rare chondrosarcoma	2004	dFFPE	Moderate	Moderate	Formic acid

9	Rare chondrosarcoma	2006	dFFPE	Moderate	Moderate	Formic acid

10	Rare chondrosarcoma	2000	dFFPE	Moderate	Moderate	Formic acid

11	Rare chondrosarcoma	1996	dFFPE	Moderate	Moderate	Formic acid

12	Rare chondrosarcoma	NA	dFFPE	High	Moderate	Formic acid

13	Rare chondrosarcoma	NA	dFFPE	High	Moderate	Formic acid

14	Rare chondrosarcoma	1994	dFFPE	High	Moderate	Formic acid

15	Rare chondrosarcoma	NA	dFFPE	High	Moderate	Formic acid

16	Rare chondrosarcoma	2007	dFFPE	High	Moderate	Formic acid

17	Rare chondrosarcoma	NA	dFFPE	High	Moderate	Formic acid

18	Rare chondrosarcoma	2001	dFFPE	High	Moderate	Formic acid

19	Rare chondrosarcoma	1994	dFFPE	High	Moderate	Formic acid

20	Rare chondrosarcoma	1996	dFFPE	High	Moderate	Formic acid

21*	Rare chondrosarcoma	2000	Frozen	Moderate	Moderate	None

22**	Rare chondrosarcoma	1996	Frozen	Moderate	Moderate	None

23	Chondrosarcoma	2001	Frozen	High	High	None

24	Chondrosarcoma	2003	Frozen	low	High	None

25	Primary angiosarcoma	2007	Frozen	High	Low	None

26	Primary angiosarcoma	NA	dFFPE	High	Low	Formic acid

27	Primary angiosarcoma	2007	dFFPE	High	Low	Formic acid

28	Primary angiosarcoma	2007	FFPE	High	Low	None

29	Primary angiosarcoma	NA	FFPE	High	Low	None

30	Primary angiosarcoma	2007	FFPE	High	Low	None

31	Primary angiosarcoma	NA	dFFPE	High	Low	Formic acid

32	Chondrosarcoma	1990	dFFPE	Low	High	Formic acid

* Corresponding frozen sample Nr 10.

** Corresponding frozen sample Nr 11.

***NA: not available

For one case (Nr 10) array comparison using DNA isolated from dFFPE tissue and the corresponding frozen tissue part was possible.

All samples were handled in a coded fashion, and all procedures were performed according to the ethical guidelines, ''Code for Proper Secondary Use of Human Tissue in the Netherlands'' (Dutch Federation of Medical Scientific Societies).

### DNA isolation

Five to ten 0.2 mm FFPE punches or two to five 20 μm thick dFFPE sections were collected depending on tissue type and tumour content. From each block a 4 μm consecutive section was cut and stained using standard haematoxylin and eosin (HE) staining to visualise target cells and served as control. An optimized DNA isolation protocol was developed based on the use of Macherey-Nagel Nucleospin Tissue kit. Briefly, sections/punches were collected into an Eppendorf tube and were deparaffinised using two cycles of xylene incubation, 15 min each at room temperature, followed by two steps of 100% ethanol incubation, 15 min each. Samples were then dried and 200 μl PK1 buffer supplemented with Proteinase K (0.4 mg/ml) was added to each tube and incubated for 18 hours at 56 °C. On day two, 200 μl buffer B3 was added to each vial. Samples were vortexed vigorously, incubated at 70°C for 10 min and vortexed again. By these means, most tissue pieces were dissolved. When visible particles were left (typically bone remnants), samples were centrifuged for 5 min at 11.000 × g and supernatant was transferred to a new tube. Before loading samples to a DNA binding column, 210 μl 100% ethanol was added. At this step, a partial precipitation within the solution was observed in some of the samples. For DNA binding, samples were centrifuged for 1 min at 11.000 × g. In some cases, repeated centrifugation steps were necessary. Flow-through was discarded and columns were washed by adding 500 μl BW solution followed by 1 min 11.000 × g centrifugation step, followed by a second wash step using 600 μl B5 buffer and centrifugation. To elute the DNA 50 μl preheated (70°C) MQ solution was added to the column and incubated at room temperature for 5 min followed by a centrifugation step at 11000 × g for 1 min.

DNA isolation from frozen tissue was performed as described earlier [6].

### Sample assessment

DNA concentrations were measured using a Nanodrop ND-1000 spectrophotometer and 500 ng was electrophoresed in a 1% agarose gel stained with ethidium bromide.

### Sample labelling

#### Agilent Oligo aCGH Labeling Kit for FFPE Samples (Agilent) utilising ULS labelling system

Labelling was done according to the manufacturer's recommendations with some modifications. In brief, for 44k Agilent arrays (Agilent Technologies, Santa Clara, CA), 500 ng DNA was chemically labelled with Universal Linkage System (ULS) Cy3 (test) or Cy5 (reference)-dyes. Before labelling, reference samples were heat fragmented in order to achieve equal fragment sizes in both test and reference sample. The labelled samples were then purified using the Agilent KREApure columns. Labelling efficiency was calculated using a Nanodrop Spectrophotometer measuring A_260 _(DNA), A_550 _(Cy3) and A_649 _(Cy5).

#### BioPrime Total FFPE Genomic Labelling System (Invitrogen)

Labelling was done according to the manufacturer's recommendations with some modifications. In brief, 500 ng DNA was used for labelling, instead of the recommended 1 μg DNA. Labelling with both 150 ng and 500 ng DNA was done for one sample (Nr 13). Random prime (RP) labelling was done by using the BioPrime Total FFPE Genomic Labelling System (Invitrogen Corporation, Carlsbad, CA) Labelling efficiency was calculated using a Nanodrop Spectrophotometer measuring A_260 _(DNA), A_550 _(Cy3) and A_649 _(Cy5). Heat-fragmented DNA from a commercial source (Promega Corporation, Madison, WI) was used as a reference. Samples were labelled with Alexa Fluor 3 mix (test sample) and Alexa Fluor 5 mix (reference sample).

For both ULS- and RP-system-labelled test and reference samples were mixed and hybridized as a gender mismatch to show dynamic range of hybridisation on the X and Y chromosomes. Two samples were labelled both with the random prime kit and with ULS (Nr 10 and Nr 18).

### Hybridisation, scanning and, data extraction

Hybridisation was performed on a 4 × 44k Agilent oligo array Chip at 65°C for 40 hours. Slides were washed with Oligo aCGH Wash Buffer 1 at room temperature for 5 min followed by a 1 min wash with Oligo aCGH Wash Buffer 2 at 37°C. Finally, slides were dried without using the stabilisation and drying solution. Slides were scanned using an Agilent Scanner with 5 μm scan resolution. Scan images were processed with Feature Extraction Software and Genomic Workbench (Agilent Technologies, Santa Clara, CA). All samples, irrespective of quality, were processed for further comparisons.

### Interphase FISH confirmation

To confirm one of the array-CGH results of case Nr 26, a two-colour interphase FISH experiment was done. A BAC-clone (RP1-80K22) located at 8q24.21 covering the *MYC *gene locus (detected in red) in combination with an alpha satellite probe specific to the centromeric region of chromosome 8 (detected in green) were used as described earlier [7].

### Statistical analysis

Log2 transformed ratio values were extracted from the scan images and processed using the Feature Extraction Software package and Genomic Workbench (Agilent). The exported log2 transformed ratio values were used for further comparison. Correlations were calculated using Pearson coefficients and systematic bias calculations were done by using Bland-Altman plots using the SPSS 16.0 for Windows software package. For the Bland-Altman plots the differences between the two individual reporters measured by two experiments on the y axis were plotted against the mean log2 ratio of the two on the x axis. This test allows the investigation of systematic bias. Relatively small differences and little bias are represented by a "flat profile". For the comparison of the resulting array-CGH profiles we used the CGHCall R script developed by van de Wiel et al.[8].

## Results

### DNA quality and quantity assessment

**Figure 1:**
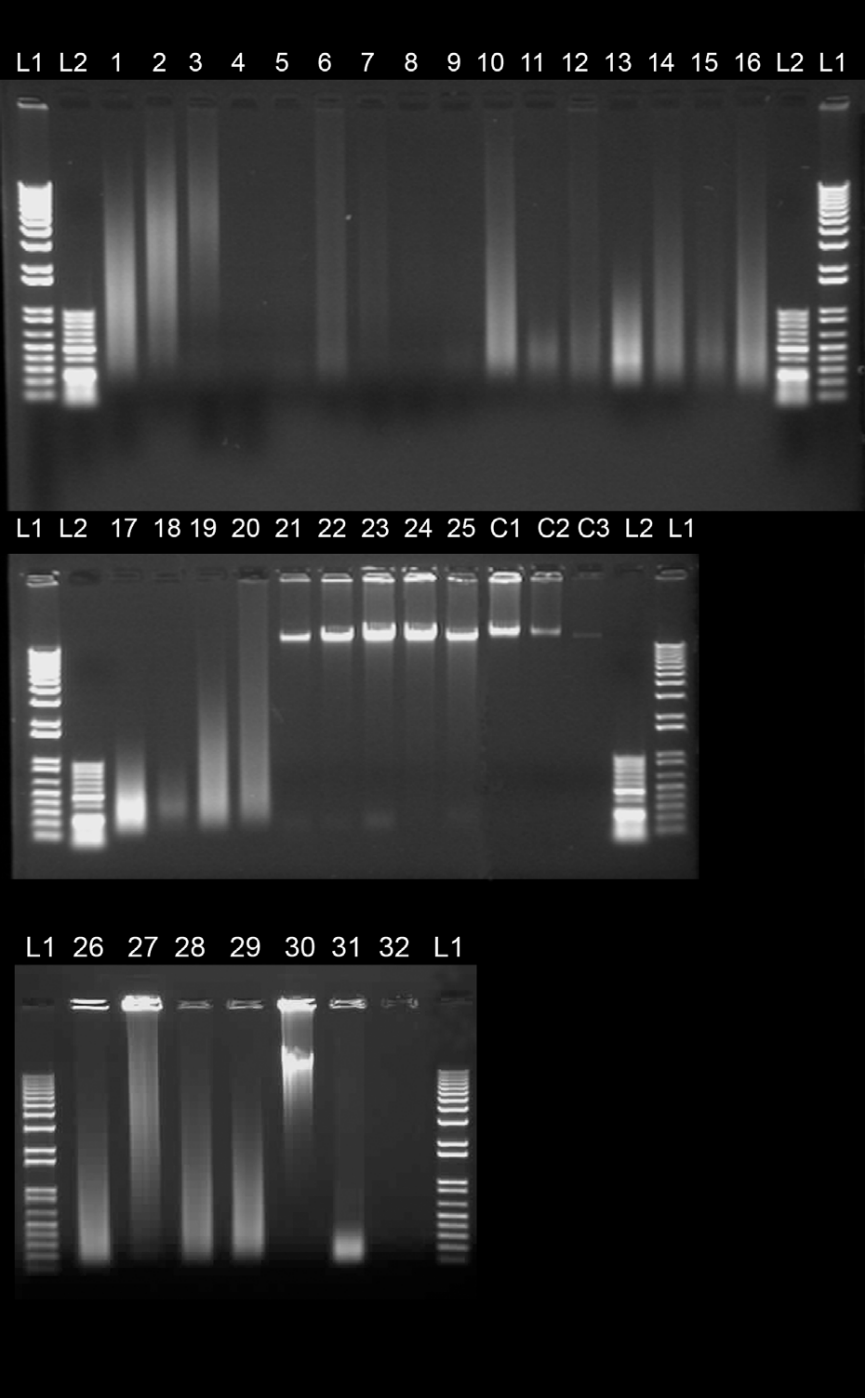
**Quality and quantity assessment of DNA samples: Image of a 1% agarose gel separation after ethidium bromide staining depicting several representative tumour samples for testing**. L1 and L2 represent 1 kb+ and 50 bp ladders, respectively. C1, C2, C3 are high molecular weight genomic DNA samples with known concentrations of 500, 250 and 50 ng, respectively. Detailed sample characteristics are provided in Table 2.

DNA concentration was estimated using the Nanodrop system and equal amounts of DNA were electrophoresed in a 1% agarose gel. The absorption based measurement using the Nanodrop system showed inconsistent results when values were compared to agarose gel images. Figure 1 shows a diverse range of DNA fragment sizes for all samples. Samples with moderate (for example nr 3 and 27) to severe (sample nr 17, and 31) DNA degradation showed acceptable CGH profiles. In general, DNA concentration was overestimated particularly for cartilaginous tumour samples with high extracellular matrix composition. In these cases relatively low concentrations were measured (typically in the range of 2-15 ng/μl) (Table 2) but determining the concentration based on the corresponding gel image suggested that these measurements were an over estimate (Figure 1) (for example: samples nr 9, 11, 15 and 18). As for all labelling reactions, the initial amount of starting material is a crucial factor. We corrected the DNA concentration measured by Nanodrop using the integral of the UV-excited ethidium bromide fluorescence obtained from the agarose gel images. For these measurements, known amounts of reference DNA samples were loaded. The correction factor between the two types of measurements, especially at the lower concentration range, was as high as 10 fold resulting in significant over estimation of sample concentration for labelling and consecutive testing.

**Table 2:** Overview of DNA concentrations using Nanodrop and Gel based estimation

Sample ID*	Nanodrop conc (ng/μl)	260/280 ratio	260/230 ratio	Gel-based conc (ng/μl)	Correction factor**	Array QC***
1	131.0	1.84	2.47	134.5	0.97	OK

2	153.0	1.84	2.42	130.7	1.17	OK

3	31.9	1.88	2.67	24.9	1.28	OK

4	10.9	1.66	0.41	4.5	2.41	NP

5	13.6	2.08	1.57	3.9	3.51	NP

6	44.7	1.8	1.38	20.5	2.18	NP

7	9.8	2.06	1.92	9.0	1.08	NP

8	44.0	1.1	0.3	3.1	14.36	NP

9	57.5	1.57	0.82	3.8	14.94	Poor

10	540.0	1.78	1.9	346.9	1.56	OK

11	164.0	1.63	1.24	19.5	8.42	Poor

12	102.0	1.76	1.98	19.3	5.27	OK

13	485.0	1.84	2.34	270.5	1.79	OK

14	291.0	1.8	2.3	127.8	2.28	OK

15	430.0	1.69	2.17	76.3	5.64	Poor

16	208.0	1.81	2.21	135.9	1.53	OK

17	269.0	1.84	2.35	192.3	1.40	OK

18	63.0	1.73	2.11	9.7	6.48	Poor

19	215.0	1.75	2.12	146.6	1.47	OK

20	285.0	1.7	2.37	170.4	1.67	OK

21	1376.0	1.78	1.28	565.6	2.43	OK

22	88.6	1.67	0.91	68.1	1.30	OK

23	259.8	1.8	1.75	286.8	0.91	OK

24	496.4	1.7	1.8	523.9	0.95	OK

25	30.6	1.7	1.6	25.2	1.22	NP

26	172.0	1.8	2.11	NP	NP	OK

27	306.0	1.75	1.98	NP	NP	OK

28	203.0	1.89	2.31	NP	NP	OK

29	132.9	1.78	2.05	NP	NP	OK

30	300.0	1.8	1.96	NP	NP	OK

31	71.0	1.67	0.89	NP	NP	OK

32	47.5	1.71	0.68	NP	NP	Poor

C1	500	1.8	1.95	462.9	1.08	NP

C2	250	1.8	1.95	238	1.05	NP

C3	50	1.8	1.95	45	1.11	NP

* Sample ID corresponds to the sample label in Figure 1

** Nanodrop concentration/Gel-based concentration

*** NP: Not performed

### Comparison of different labelling approaches

**Figure 2:**
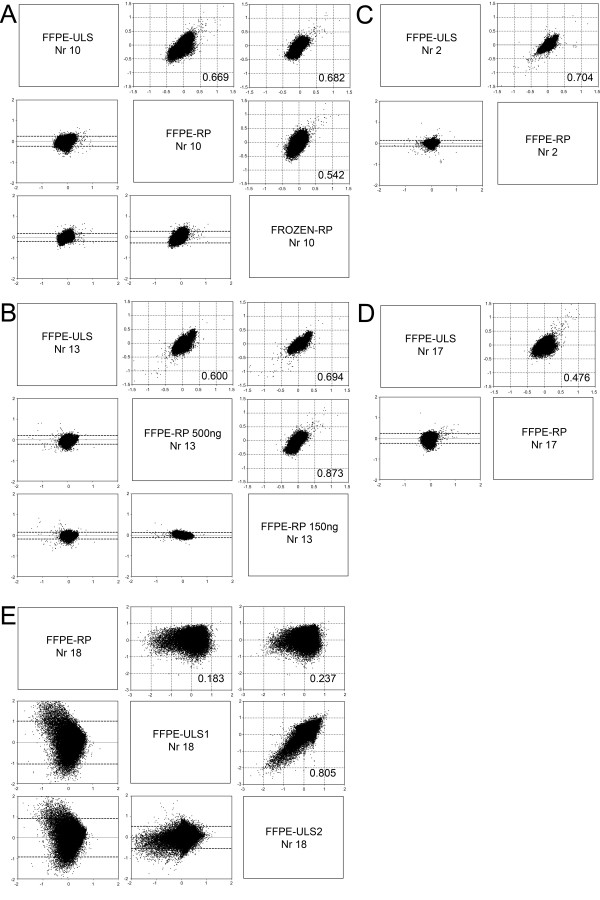
**Array-CGH plots of decalcified FFPE samples after ULS or RP labelling**. For all plots: Upper right: Correlation plots of log2 ratios for each reporter between experiments, with linear regression and Pearson's correlation coefficients given. Lower left: Bland-Altman plots of the differences between two reporters measured by two experiments on the y axis against the mean log2 ratio of the two on the x axis. A: Correlation plots of sample Nr 10 comparing hybridisation of random prime and ULS based labelling of FFPE and RP labelling of frozen tissue derived DNA samples. B: Correlation plots of sample Nr 13 using FFPE isolated DNA samples with ULS, 150 ng RP and 500 ng RP labelling. C, D Correlation plots of samples Nr 2 and Nr 17 using FFPE isolated DNA samples with ULS or RP labelling reactions. E: Correlation plot of sample Nr 18. This sample showed a great degree of discrepancy for the estimated DNA concentration between the absorption based and the gel based measurements.

**Figure 3:**
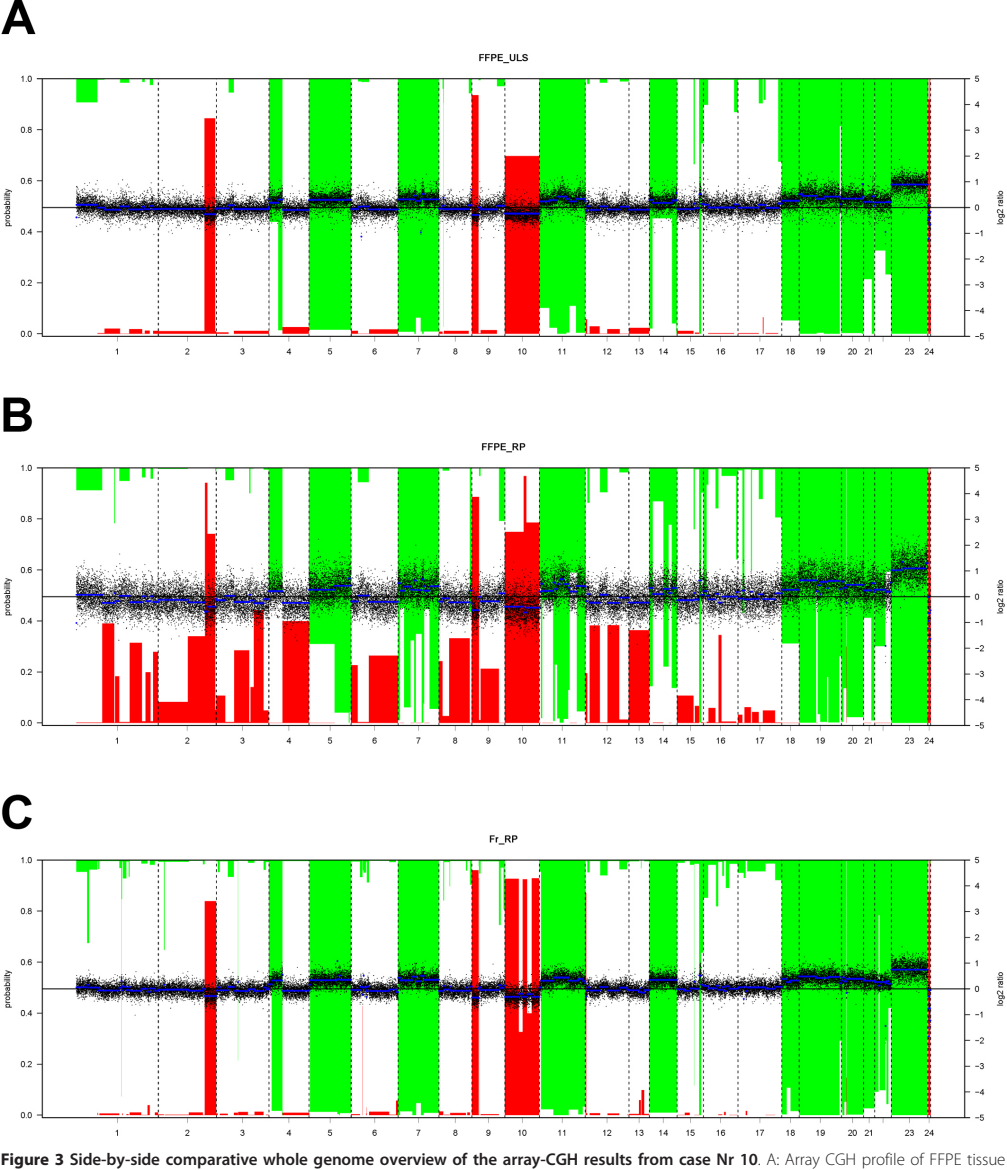
**Side-by-side comparative whole genome overview of the array-CGH results from case Nr 10**. A: Array CGH profile of FFPE tissue isolated DNA sample after ULS labelling (FFPE-ULS). B: Array CGH profile of FFPE tissue isolated DNA sample after using a Random Primer labelling especially designed to label FFPE samples (FFPE-RP). C: Array-CGH profile of frozen tissue isolated DNA after standard Random Prime Labelling reaction (Fr-RP). Normalized log2-ratios are plotted with the scale on the right axis. Vertical bars indicate loss and gain probabilities. Probability scale is on the left axis; reversed ('1-') for the gains. Segments are plotted as horizontal blue lines. Segments with a bar extending beyond the middle axis (probability >0.5) are called as gain or losses. All plots were generated using the CGHCall R software package. The variation of the reporter signals was the highest (black dots represent individual reporter elements) in case of FFPE-RP labelling (see also Figures 2a) followed by FFPE-ULS and Fr-RP. In all three profiles almost identical calls were present (see Table 3 for details on the called regions).

Different comparisons were made based on the type of samples available. A three-way comparison was made for Nr 10 with DNA collected from both frozen and dFFPE material. DNA from frozen tissue was labelled using a random primer labelling kit and DNA from dFFPE tissue was labelled with both the random primer labelling kit designed for FFPE samples and ULS labelling kit for FFPE samples (Figure 2A, Figure 3). The different labelling schemes showed an overall good correlation, the Pearson correlation coefficient varied between 0.542 and 0.682 and showed a better correlation between the ULS-FFPE vs RP frozen (0.682) than the RP frozen vs RP-FFPE reaction (0.542). Very good agreement was observed between the two different labelling reactions using dFFPE samples (0.669). Side-by-side comparative whole genome overview of the array-CGH results showed the variation of the reporter signals was highest (black dots represent individual reporter elements) in the case of FFPE-RP labelling, followed by FFPE-ULS and Fr-RP. In all three profiles almost identical aberrations were present (see Table 3 for an overview of the genome-wide genomic aberrations).

**Table 3:** Overview of genome-wide genomic aberrations of dFFPE sample Nr 10 and the corresponding frozen sample

Chr	Cyto band	Start (bp)*	Stop (bp)*	Nr of Probes	Gain (+)/ Deletion (-)	FrozenRP	dFFPERP	dFFPEULS
1	p36.33 - p33	749422	46786807	1053	+	+	+	+

1	p12 - q23.3	119416284	160645328	539	+	NC**	+	+

2	q33.2 - q37.3	204593489	242169652	575	-	+	+	NC**

4	p16.3 - q13.3	146653	70631034	683	+	+	+	+

5	p15.33 - q35.3	1163403	180617248	2104	+	+	+	+

6	p22.1 - p21.1	26128906	44328148	558	+	NC**	+	+

7	p22.3 - q36.3	289341	158602640	2056	+	+	+	+

9	p24.3 - p13.3	322256	33155616	370	-	+	+	+

9	q33.3 - q34.3	129159725	140128884	293	+	NC**	+	+

10	p15.3 - q26.3	138006	135222624	1739	-	+	+	+

11	p15.4 - q25	2906039	133951511	2213	+	+	+	+

12	q13.11 - q14.1	47340134	56637091	379	+	+	+	+

14	q11.2 - q32.33	19508645	106330010	1394	+	+	+	+

15	q25.3 - q26.1	86577905	91761128	104	+	+	+	+

16	p13.3 - q24.3	36566	88572953	1741	+	+	+	+

17	p13.3 - q25.3	295150	78154619	2163	+	+	+	+

18	p11.32 - q23	170029	76083258	875	+	+	+	+

19	p13.3 - q13.43	231880	63389940	2096	+	+	+	+

20	p13 - q13.33	73854	62363774	1115	+	+	+	+

21	p11.1 - q22.3	10013063	46646924	549	+	+	+	+

22	q11.1 - q13.33	14433273	49525271	833	+	+	+	+

22	q13.1	37688858	37715585	3	-	+	NC**	+

* according to NCBI hg18

** NC: not called

Since for routine applications the amount of DNA for testing is often limited, we compared the influence of lower amounts of starting material for labelling using 500 ng and 150 ng dFFPE-isolated DNA for the FFPE-RP kit (Nr 13). These results were compared to ULS labelling reaction using 500 ng of DNA (Figure 2B). Based on the comparison of the overall profiles, the best correlation was observed between the two FFPE-RP reactions (0.873) using different input for labelling (150 vs 500 ng dFFPE DNA for FFPE-RP kit) followed by a 0.694 between the 500 ng FFPE-ULS and 150 ng FFPE-RP. This correlation shows that the type of labelling bias, introduced by the labelling kit of choice, makes the overall profile more alike suggesting that ULS labelling of samples will result in a comparable profile of other ULS samples while the FFPE-RP kit will have its own bias and similar profiles for comparison between different samples. In contrast to this, the influence of sample storage (FPPE vs frozen) was stronger than the influence of labelling kit used (FFPE-RP or frozen-RP vs ULS) as we observed better correlation between the independent labelling reaction (ULS, FFPE-RP) than between the frozen RP and FFPE-RP labelling reaction (Figure 2A).

Poor correlations and corresponding array profiles were seen for samples with very low amounts of DNA irrespective of labelling reactions (Figure 2E and samples 9, 11, 15 and 18 in Table 2). For these reactions a minimum of 50 to 100 ng DNA was used. These results indicate the possible presence of substances influencing the efficiency of both the chemical and enzymatic labelling reactions. Because of the poor array performance using very low DNA concentrations (samples 9, 11, 15 and 18), five other samples with similarly low DNA concentrations (samples 4, 5, 6, 7, and 8) were not tested as indicated in Table 2.

For sample nr 2 and nr 17 the Pearson correlation coefficient varied between 0.704 and 0.476 (Figure 2C and 2D), respectively. Despite the weaker correlation for case 17, both arrays showed similar profiles and similar gains and losses were detected.

### Interphase FISH

**Figure 4:**
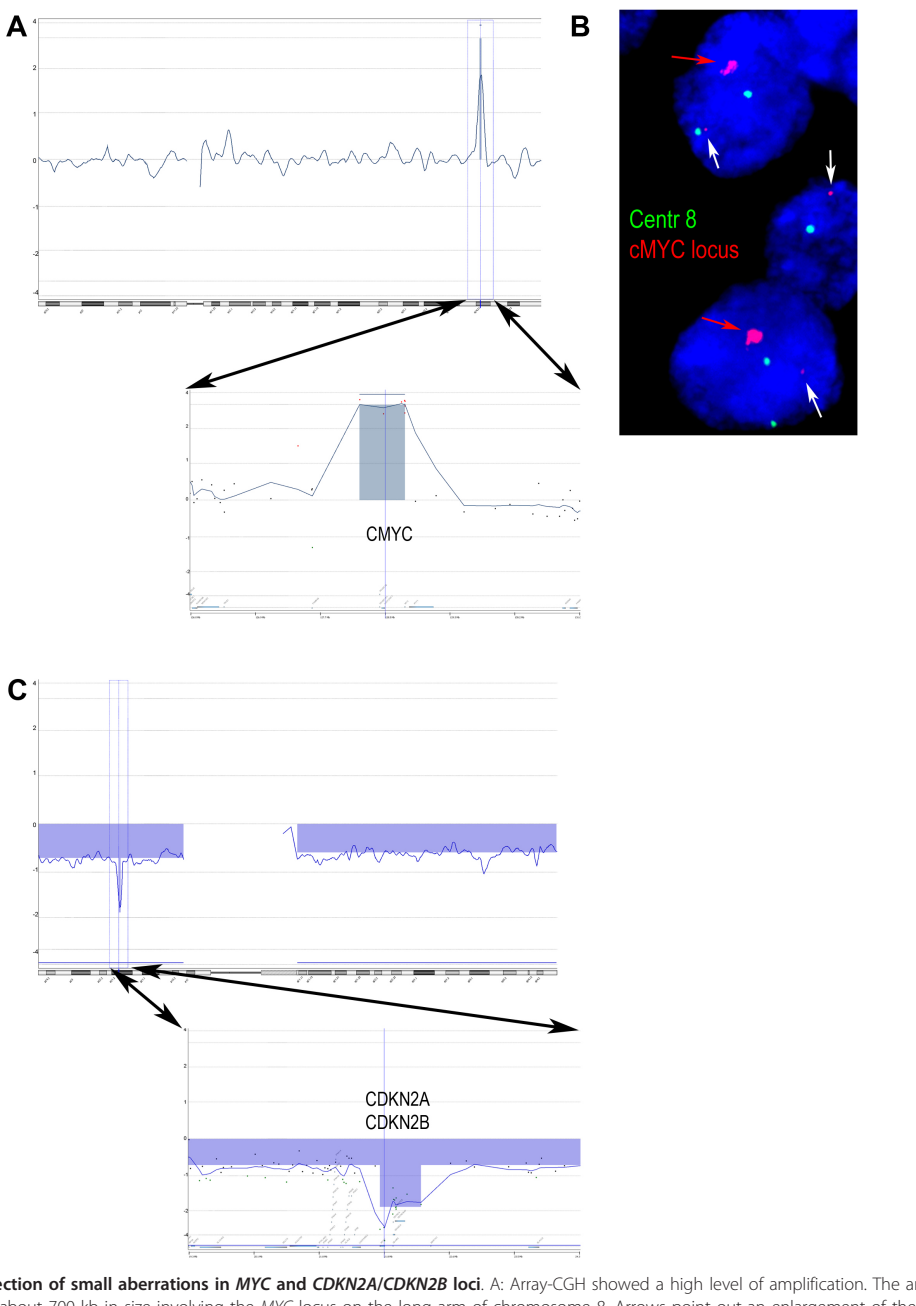
**Detection of small aberrations in *MYC *and *CDKN2A*/*CDKN2B *loci**. A: Array-CGH showed a high level of amplification. The amplified region of was about 700 kb in size involving the *MYC *locus on the long arm of chromosome 8. Arrows point out an enlargement of the *MYC *locus. B: Interphase FISH verification using chromosome 8 centromere specific probe (green) and *MYC *locus specific BAC probe (red) on the corresponding dFFPE section. The red arrow indicates signals of the amplified *MYC *locus and white arrow points to the normal locus. C: Array-CGH result of a case with homozygous deletion. The estimated size of the homozygous deleted area was about 600 kb involving the *CDKN2A/CDKN2B *loci on the short arm of chromosome 9. Arrows point out the region of the homozygous deletion, containing the *CDKN2A/CDKN2B *loci.

In two different samples, we readily detected a high level of amplification of the *MYC *locus and a homozygous deletion of the *CDKN2A/CDKN2B *loci with estimated sizes of 0.7 Mb and 0.6 Mb, respectively (Figure 4A, C).

The two-colour Interphase FISH performed on dFFPE tissue sections of case 26 showed a significant increase of signal involving BAC-clone RP1-80K22 on one chromosome arm only (Figure 4B). This pattern is compatible with the amplification of the *MYC *locus as was detected by the array-CGH test using the corresponding dFFPE tissue isolated DNA.

## Discussion

We have established and successfully applied a robust protocol to study heavily degraded DNA, obtained from decalcified FFPE samples, collected from various institutions using an oligonucleotide-based chip platform.

Both formic acid based decalcification and fixation with non-buffered formalin solution similarly degrade tissue DNA. As the average fragment length of the DNA obtained from these samples is often less than 200 bps, these are regarded as unsuited for further molecular DNA testing [4,5,9].

In this study we used oligonucleotide based array chips containing reporter elements of ~60 bps. For optimal hybridisation the fragment length of the labelled DNA sample should be similar in size as the reporter elements (60-150 bps) [3]. Because enzymatic labelling is introducing further fragmentation during labelling, we applied the Universal Linkage System (ULS) labelling technology, which is a direct chemical labelling, without introducing further fragmentation [9,10]. In addition, we compared the ULS labelling system to a commercially available random primer (RP) labelling kit especially developed for FFPE tissue derived DNA.

The overall reproducibility of the two FFPE labelling systems tested was excellent (Figure 2). With both kits we were able to obtain good results using 500 ng of starting material in contrast to the 1 μg DNA recommended by the vendors. The RP labelling has the benefit of amplifying the samples during the labelling reaction. By using as little as 150 ng degraded dFFPE DNA template for the reaction, we obtained similar results to using 500 ng (Figure 2B). However, further reduction of the starting material, especially in cases with discrepancies between estimated DNA concentrations in different measuring methods, resulted in poor results. The use of less than 500 ng DNA for ULS labelling resulted in too weak signals and is therefore not recommended.

Samples labelled with the RP kit showed higher fluorescence intensities after scanning as compared to the ULS labelled samples. However, the overall variance of the log2 ratio distribution of the signal was higher as compared to the ULS system (Figure 3A, B). For one case (Nr 10), we had access to both frozen and dFFPE samples. By comparing three kinds of labelling systems a good correlation was observed between all labelling systems and samples (Figure 3, Table 3).

We showed that, irrespective of the fragment size of the DNA, all samples with sufficient quantity were eligible for testing. Since correctly estimated DNA concentration is more critical for successful testing than the quality of the DNA (i.e. fragment size), DNA concentrations were established by using two independent approaches. For some samples we observed discrepancies between the absorption-based DNA concentration measurement and the estimation based on ethidium bromide stained gel imaging. In general, the absorption based system tends to overestimate the final DNA yield resulting in a suboptimal amount for testing (Figure 1). This observed difference might, in part, be explained by the presence of negatively charged matrix glycoproteins such as chondroitin 4-sulphate, chondroitin 6-sulphate and keratan sulphate in some of the tumour samples. Some of these matrix glycoproteins may have similar charges as DNA and consequently could bind to the purification columns when the total DNA content of the sample was low. None of the used labelling systems gave reliable array profiles in cases with high over estimates of concentration. In these cases, in addition to the low DNA concentration, other factors might interfere with the labelling reaction and could be responsible for the failure.

The low amount of DNA might be compensated for by a whole genome amplification step using DOP-PCR, GenomePlex or Phi29 polymerase based reactions. However, it has been shown by others that when using good quality FFPE samples, DOP-PCR results in amplification biases and GenomePlex was suitable in only 58% of the analysed cases [9,11]. The use of multiplex PCR based pre-screening of FFPE samples may be used to select samples, however, it is noteworthy that most of our samples were degraded beyond the exclusion limits of those QC reactions and would not provide a good prediction [4,5,9]. There are several reports using FFPE samples for genomic profiling either on BAC array [4], oligonucleotide based array or the Illumina Golden Gate SNP array systems [6,12,13]. The Golden Gate system has a relatively low resolution consisting of approximately 6000 SNP reporter elements with an average physical distance of about 500 kb. Due to the increased variation of signal ratio values, extensive smoothing steps (i.e. averaging of multiple probes for a given segment) are routinely applied to even out these variations. In turn, the overall resolution of these platforms decreases and most of the changes reported will concern whole chromosome arms or chromosome regions over at least 15-20 Mb in size. In contrast to these limitations, the procedure we established readily detected both homozygous deletions and high level of amplifications of 0.6 and 0.7 Mb in size, respectively (Figure 4).

## Conclusions

We developed a reliable DNA isolation and labelling procedure using decalcified, formalin-fixed, paraffin-embedded tissue from various clinical specimens. Using two independent techniques (gel-based and absorption-based), we showed that the estimation of DNA concentration is a more critical step in sample quality assessment than DNA quality (assessed by the degree of fragmentation). In our assessment, both the direct-chemical-labelling-based ULS kit and the modified random-prime labelling kit worked equally well.

## Competing interests

The authors declare that they have no competing interests.

## Authors' contributions

DJ conceived of the study, participated in the microarray experiments and analyses and drafted the manuscript, DM participated in the microarray experiments, sample selection and preparation, SLJV participated in the microarray experiments, sample selection and preparation, PCWH participated in the design and coordination of the study and helped to draft the manuscript, JVMGB participated in the design and coordination of the study and helped to draft the manuscript, KS carried out the microarray analyses, conceived of the study, participated in its design and coordination and helped to draft the manuscript. All authors read and approved the final manuscript.

## References

[B1] Speicher MR Carter NP The new cytogenetics: blurring the boundaries with molecular biology Nat Rev Genet 2005 6 782 792 10.1038/nrg1692 16145555

[B2] Kallioniemi A CGH microarrays and cancer Curr Opin Biotechnol 2008 19 36 40 10.1016/j.copbio.2007.11.004 18162393

[B3] Ylstra B van den Ijssel P Carvalho B Brakenhoff RH Meijer GA BAC to the future! or oligonucleotides: a perspective for micro array comparative genomic hybridization (array CGH) Nucleic Acids Res 2006 34 445 450 10.1093/nar/gkj456 16439806 PMC1356528

[B4] van Beers EH Joosse SA Ligtenberg MJ Fles R Hogervorst FB Verhoef S Nederlof PM A multiplex PCR predictor for aCGH success of FFPE samples Br J Cancer 2006 94 333 337 10.1038/sj.bjc.6602889 16333309 PMC2361127

[B5] Buffart TE Tijssen M Krugers T Carvalho B Smeets SJ Brakenhoff RH Grabsch H Meijer GA Sadowski HB Ylstra B DNA quality assessment for array CGH by isothermal whole genome amplification Cell Oncol 2007 29 351 359 17641418 10.1155/2007/709290 PMC4617808

[B6] Oosting J Lips EH van Eijk R Eilers PH Szuhai K Wijmenga C Morreau H van Wezel T High-resolution copy number analysis of paraffin-embedded archival tissue using SNP BeadArrays Genome Res 2007 17 368 376 10.1101/gr.5686107 17267813 PMC1800928

[B7] Rossi S Szuhai K Ijszenga M Tanke HJ Zanatta L Sciot R Fletcher CD Dei Tos AP Hogendoorn PCW EWSR1-CREB1 and EWSR1-ATF1 fusion genes in angiomatoid fibrous histiocytoma Clin Cancer Res 2007 13 7322 7328 10.1158/1078-0432.CCR-07-1744 18094413

[B8] Van De Wiel MA Kim KI Vosse SJ Van Wieringen WN Wilting SM Ylstra B CGHcall: calling aberrations for array CGH tumor profiles Bioinformatics 2007 23 892 894 10.1093/bioinformatics/btm030 17267432

[B9] Knijnenburg J van der Burg M Tanke HJ Szuhai K Optimized amplification and fluorescent labeling of small cell samples for genomic array-CGH Cytometry A 2007 71 585 591 17458882 10.1002/cyto.a.20412

[B10] Raap AK van der Burg MJ Knijnenburg J Meershoek E Rosenberg C Gray JW Wiegant J Hodgson JG Tanke HJ Array comparative genomic hybridization with cyanin cis-platinum-labeled DNAs Biotechniques 2004 37 130 134 15283211 10.2144/04371DD03

[B11] Little SE Vuononvirta R Reis-Filho JS Natrajan R Iravani M Fenwick K Mackay A Ashworth A Pritchard-Jones K Jones C Array CGH using whole genome amplification of fresh-frozen and formalin-fixed, paraffin-embedded tumor DNA Genomics 2006 87 298 306 10.1016/j.ygeno.2005.09.019 16271290

[B12] Lips EH de Graaf EJ Tollenaar RA van Eijk R Oosting J Szuhai K Karsten T Nanya Y Ogawa S van de Velde CJ Single nucleotide polymorphism array analysis of chromosomal instability patterns discriminates rectal adenomas from carcinomas J Pathol 2007 212 269 277 10.1002/path.2180 17471469

[B13] Buffart TE van Grieken NC Tijssen M Coffa J Ylstra B Grabsch HI Van d V Carvalho B Meijer GA High resolution analysis of DNA copy-number aberrations of chromosomes 8, 13, and 20 in gastric cancers Virchows Arch 2009 455 213 223 10.1007/s00428-009-0814-y 19697059 PMC2744787

